# Periodontal health among chartered accountants in India

**DOI:** 10.6026/973206300211231

**Published:** 2025-05-31

**Authors:** Eshita Patel, Hiral Akshat Parikh, Patel Patel, Colleen A. Watson, Jay Soni, Shrikant Patel

**Affiliations:** 1 Department of Periodontology, AMC Dental College and Hospital, Khokhra, Ahmedabad, Gujarat, India; 2 Department of Periodontology, Narsinhbhai Patel Dental College & Hospital, Sakalchand Patel University, Visnagar, Gujarat, India; 3 Department of Dental Surgery, Pacific Dental College, Udaipur, Rajasthan, India; 4 Department of General Dentistry and Comprehensive Care, NYU College of Dentistry, New York, USA; 5 Department of Cariology and Comprehensive Care, NYU College of Dentistry, New York, USA

**Keywords:** Chartered accountant, periodontal awareness, periodontal health

## Abstract

Periodontal awareness and the impact of lifestyle on periodontal health among Chartered Accountants (CAs) in India is of interest. A cross-sectional study was conducted among 514 CAs aged 20 and above. An online survey evaluating demographics, oral hygiene knowledge and lifestyle was administered to the patients. Participants showed moderate awareness of periodontal health, while 62.84% recognized link between oral and general health, over a third were unaware or unsure, highlighting the need for increased awareness. This study highlights a moderate level of periodontal awareness among Chartered Accountants in Ahmedabad.

## Background:

Oral health is a key indicator of general health, reflecting overall well-being and systemic conditions [1, 2]. The FDI (2016) defines oral health as the ability to chew, speak and express without pain or disease, influenced by social, biological and environmental factors [3]. Periodontal diseases are among the most widespread globally and in densely populated countries like India, their burden is especially high [1, 2]. Poor oral hygiene, lifestyle habits and systemic conditions such as diabetes, poor nutrition and tobacco use contribute to the onset and progression of periodontal disease, potentially leading to tooth loss if untreated [2]. Moreover, periodontitis has been linked to systemic diseases including cardiovascular, respiratory and pregnancy-related complications [3]. Its classification as a lifestyle-related disorder highlights the importance of modifiable behaviors like smoking and diet [4, 5]. Lifestyle-shaped by culture and habits like exercise, sleep and nutrition-directly impacts oral health and can be assessed using tools like the health-Promoting Index. Raising oral hygiene awareness and early screening using indices like the CPITN are vital for prevention and care [6]. Socioeconomic and demographic factors also influence oral health outcomes [7]. Despite several epidemiological studies in India, data on oral health among Chartered Accountants (CAs) is lacking. Given their sedentary and high-stress lifestyle, this group may be at elevated risk. Therefore, it is of interest to evaluate periodontal awareness among CAs in India.

## Materials and Methods:

This cross-sectional study was conducted among 514 Chartered Accountants registered with the ICAI, Ahmedabad branch, to assess periodontal awareness on oral health. Participants aged 20 years and above who provided informed consent and completed the online survey were included. Those who were unwilling or who failed to submit the survey were excluded. An online questionnaire was distributed via Google Forms, covering demographics and periodontal knowledge. A single examiner carried out all assessments to ensure uniformity. Sterilization was maintained using a 10% Korsolex solution, with instruments submerged for 30 minutes, rinsed and reprocessed. Data was analyzed using SPSS version 15. Descriptive statistics, chi-square tests and multiple logistic regressions were applied, with significance set at p < 0.05 and a confidence level of 95%.

## Results:

Table 1 shows that most participants (49.61%) were aged 21-30 years, with only 6.22% above 50, indicating a predominantly young professional group. Table 2 reveals that 47.47% had never visited a dentist and just 8.94% did so every three months, reflecting a gap in preventive dental care. Table 3 shows that 69.06% brushed once a day, 28.79% brushed twice a day and 2.14% brushed after every meal. Table 4 indicates that while 62.84% recognized the link between oral and general health, over a third were unaware or unsure, highlighting the need for increased awareness.

## Discussion:

The WHO defines health as a complete state of well-being, with oral health playing a vital role in overall quality of life [1, 2-3]. Periodontal disease is multifactorial, influenced by plaque, systemic conditions and lifestyle habits like smoking and poor nutrition [4, 5-6]. WHO oral health policy promotes integrating oral and general health strategies by addressing shared, modifiable risk factors [7]. However, limited data exists on oral health awareness among high-stress groups like Chartered Accountants (CAs), prompting this study. Most participants were aged 21-40 and 74.7% were male. Although brushing is the most common method for cleaning teeth, this survey showed that 69.06% brushed once a day and 2.14% brushed after every meal whereas 28.79% brushed twice a day, which is same as compared to 23% of the population of Jodhpur in a study by Jain *et al.* 2012 [8] 29.6% of Maharashtra population in a study by Priyanka *et al.* 2018 [2] and 29.8% of health care workers of Lucknow in a study by Omveer *et al.* 2022 [9]. This indicates moderate compliance with oral hygiene guidelines. Masanobu Abe *et al.* found that students who brushed their teeth "twice," the risk of gum bleeding was 1.45 (1.27-1.67) times higher than that for those who brushed their teeth "three times or more" [10, 11, 12, 13-14].

Only one-third used the correct brushing technique. Around 62.84% participants knew oral health is related to general health, 26.26% believed it was not related, 5.83% were not sure and 5.05% did not know about the same in this study. In a study done by Kapoor, *et al.* 2014 only 43.2% had the knowledge that there is a relationship between the oral health and systemic health [15, 16, 17- 18]. Taking into consideration the above findings, many more studies including larger population should be conducted to evaluate and help the society for improving oral health. The association of lifestyle with periodontitis supports the concept that behavior should be taken into consideration for the prevention of chronic diseases. Negating orally-abusive substance addiction, having breakfast every day, eating a balanced diet, and reducing stress lead to an overall good lifestyle. These factors along with sleeping a minimum of 7-8 h per night and working for 8-9 h and ample daily exercise may help patients improve or protect their oral health for years to come [18].

## Source of Funding:

There was no financial support concerning this work.

## Conclusion:

No such survey has been carried out till date amongst the Chartered Accountants and hence this is the first survey of its kind amongst the CA group in India. Within the scope of this study, it was affirmed that the subjects had fair knowledge of periodontal health. Subjects were in need of increasing periodontal awareness and modify their lifestyle to certain extent to help improve their dental and overall health.

## Figures and Tables

**Table 1:** Age-wise distribution of study participants

**Age in years**	**Number**	**Percentage (%)**
21-30	255	49.61
31-40	160	31.12
41-50	67	13.03
51-60	21	4.08
More than 60	11	2.14
Total	514	100

**Table 2:** Frequency of dental visits

**Dental Visit Frequency**	**Number**	**Percentage (%)**
Every 3 months	46	8.94
Every 6 months	79	15.34
Once a year	145	28.21
Never	244	47.47
Total	514	100

**Table 3:** Distribution of study participants according to brushing frequency

**Tooth-brushing frequency**	**Number**	**Percentage (%)**
Once	355	69.06
Twice	148	28.79
After every meal	11	2.14
None	0	0
Total	514	100

**Table 4:** Awareness of oral health related to general health

**Response**	**Number**	**Percentage (%)**
Yes	323	62.84
No	135	26.26
Maybe	30	5.83
Don't know	26	5.05
Total	514	100
